# The Breast: Comprehensive Management of Benign and Malignant Disorders

**DOI:** 10.1038/sj.bjc.6602192

**Published:** 2004-10-26

**Authors:** R Sainsbury

**Affiliations:** 1 Royal Free and University College, Medical School London, UK

This is now the third edition of a book first published in 1991. The book has grown to two volumes with 30% more information than the second edition. Additional chapters include: The Management of Breast Pain and the Risk of Breast Cancer After HRT. The evolution of Sentinel Lymph Node Biopsy has been addressed and the chapter on Legal Issues Relating to Management of Breast Disease has been expanded.

One particularly welcome change has been to adopt an intemational perspective on the references rather than the usual United States-Centric list of references. The text is generally well written, readable and comprehensive, but I found the quality of the reproductions poor and the type face not the clearest to read. This is a shame as it detracts from what is otherwise a good book.

All those treating breast disease will wish to have one of the major text books on their shelf. Whether they choose this or the alterative Diseases of the Breast will be a matter of personal choice and pocket. This book is certainly worth considering and had it not been for the poor quality of the illustrations, would be my first choice.

